# Chitin-Based Anisotropic Nanostructures of Butterfly Wings for Regulating Cells Orientation

**DOI:** 10.3390/polym9090386

**Published:** 2017-08-23

**Authors:** Abdelrahman Elbaz, Jie Lu, Bingbing Gao, Fuyin Zheng, Zhongde Mu, Yuanjin Zhao, Zhongze Gu

**Affiliations:** 1 State Key Laboratory of Bioelectronics, School of Biological Science and Medical Engineering, Southeast University, Nanjing 210096, China; chem.egy@gmail.com (A.E.); 101101546@seu.edu.cn (J.L.); 230139435@seu.edu.cn (B.G.); 230139156@seu.edu.cn (F.Z.); 230129294@seu.edu.cn (Z.M.); yjzhao@seu.edu.cn (Y.Z.); 2 National Demonstration Center for Experimental Biomedical Engineering Education, Southeast University, Nanjing 210096, China; 3 Laboratory of Environment and Biosafety, Research Institute of Southeast University in Suzhou, Suzhou 215123, China

**Keywords:** butterfly wings, anisotropic nanostructure, chemical treatment, cell alignment

## Abstract

In recent years, multiple types of substrates have been applied for regulating cell orientation. Among them, surface topography patterns with grooves or ridges have been widely utilizing for cell culturing. However, this construction is still complicated, low cost-effective and exhibits some technological limitations with either “top-down” or “bottom-up” approaches. Here, a simple and green method was developed by utilizing butterfly wings (*Morpho menelaus*, *Papilio* *ulysses telegonus* and *Ornithoptera croesus lydius*) with natural anisotropic nanostructures to generate cell alignment. A two-step chemical treatment was proposed to achieve more hydrophilic butterfly wings preceding cell culturing. Furthermore, calcein acetoxymethyl ester (Calcein-AM) staining and Methylthiazolyldiphenyl-tetrazolium bromide (MTT) assay results demonstrated the appropriate viability of NIH-3T3 fibroblast cells on those butterfly wings. Moreover, the cells displayed a high degree of alignment in each specimen of these wings. We anticipate that those originating from natural butterfly wings will pose important applications for tissue engineering.

## 1. Introduction

Cell alignment plays a critical role during embryonic development, proliferation, differentiation, wound healing, and even pathological processes [1,2,3,4,5]. Therefore, the capability to generate cell alignment is of significant importance for numerous biological researches [6,7,8,9,10,11]. Now, multiple types of substrates have been applied for regulating cell orientation [12,13,14,15,16,17,18]. Among them, the surface topography pattern with grooves or ridges has been widely employed by top-down approaches, such as photolithography, inkjet printing, and etching [19,20,21,22,23,24,25]. However, the high cost, low time efficiency, and some technological limitations of these approaches have restricted their application [26,27,28,29,30,31]. Recently, bottom-up approaches, which have the advantage of low costs, versatility, and not being restricted by nanoscale dimensions, have gained increasing attention [32,33,34,35]. In fact, many natural 3D photonic architectures exist, which have drawn the attention of researchers [36,37,38,39]. For example, *Morpho* butterfly wings have been used as a vapor sensor, and Surface Enhanced Raman spectroscopy is based on its anisotropic nanostructure [40,41,42,43,44,45]. Moreover, it is well known that butterfly wings are made of chitininous structures, which have been widely used in the field of tissue engineering [46,47,48,49,50].

Herein, in this paper, a simple, cost-effective, and green method was planned and utilized in conjunction with the butterfly wings with their natural 3D microstructure, which for generating high degree of cell orientation. For comparison, three types of butterfly wings-*Morpho menelaus* (*M. menelaus*), *Papilio ulysses telegonus* (*P. u. telegonus*), and *Ornithoptera croesus lydius* (*O. c. lydius*) were selected as they belong to different genuses and possess diverse micro/nanostructures. The two-step chemical treatment was employed in order to obtain more hydrophilic wings prior to cell culturing. Cells cultured on those substrates revealed a high degree of alignment along the direction of the anisotropic structures. We anticipate that these natural substrates will provide important applications in tissue engineering.

## 2. Experimental Section

### 2.1. Materials

*M. menelaus*, *P. u. telegonus* and *O. c. lydius* were purchased from Dieyu Company (Shanghai, China). Hydrochloric acid (HCl) and sodium hydroxide (NaOH) were purchased from Aladdin Reagent (Shanghai, China). All solvent and reagents were of analytical grade, and were used without further purification. Double-distilled water was used for all experiments.

### 2.2. Apparatus

Plasma cleaner (DT-01; SZ-Omega Ltd., Suzhou city, China) was used to hydrophilize the butterfly wings. Field emission scanning electron microscopy (FESEM, Zeiss Ultra Plus; Zeiss, Jena, Germany), equipped with a JED2300 energy-dispersive X-ray spectrometer (EDS) system, and scanning electron microscopy (SEM, S-3000N; Hitachi, Tokyo, Japan) were used to observe the wing micro-/nanostructure and the morphology of the cells cultured on the wings.

### 2.3. Methods

#### 2.3.1. Treatment of Butterfly Wings

As butterfly wings are strongly hydrophobic on both the dorsal and ventral sides, we used the plasma cleaner to hydrophilize them. Briefly, the scale lumen was pumped into a vacuum of 10 Pa, and then flushed by air to a pressure of 60 Pa. After 120-s plasma treatment at 150 W, hydroxyl (–OH) was generated on the wings, rendering them hydrophilic. Both sides of the wings were hydrophilized to avoid the wings floating on the surface of aqueous solutions and hindering subsequent experiments. To alter the main wing compound chitin to chitosan, the wings were soaked in 1 M HCl for 2 h at room temperature to remove any unwanted contaminations which came from the butterfly living environment. After rinsing with double-distilled water to remove the consumed HCl, the wings were soaked in 2 M NaOH for 30 min. Then, the NaOH solution was heated to 80 °C (*M. menelaus*, 6 h; *P. u. telegonus* and *O. c. lydius*, 12 h). Then, the wings were rinsed with double-distilled water twice to remove the remaining NaOH.

#### 2.3.2. Cell Culture

NIH-3T3 fibroblast cells were cultured in high-glucose Dulbecco’s modified Eagle’s medium (High Glucose DMEM; Invitrogen, Nanjing and Biotech Development Co., Ltd., Nanjing, China), supplemented with 10% fetal bovine serum, including 2.5 mM l-glutamine, 15 mM HPES, 0.5 mM sodium pyruvate, and 1.2 g/L sodium bicarbonate, and were supplemented with a 0.4 mg/mL G418 antibiotic. The wings (substrates) were sterilized by 75% ethanol and 1-h ultraviolet irradiation, and were placed into six-well culture plates. Sterilized metal rings were used to fix the substrate in the well and focus on the cultured areas, and the cells were cultured at a density of 1.5 × 10^6^ cells/ and incubated at 37 °C in 5% CO_2_. The culture medium was changed every two days until the cells were imaged and underwent other assessments.

#### 2.3.3. Tetrazolium (MTT) Viability Assay

Cells were cultured on the wings for 48 h (acute toxicity) or for 2 h-21 days (chronic toxicity). MTT (3-(4,5-dimethylthiazol-2-yl)-2,5-diphenyl tetrazolium bromide; Sigma, St. Louis, MO, USA) assay was applied to measure the cell viability, where the tetrazolium ring is cleaved by mitochondrial dehydrogenase enzymes to form a purple precipitate. MTT (0.5 mg/mL) was added to cells in Dulbecco’s modified eagle’s medium (DMEM) without phenol red. After 1 h incubation, the purple precipitate was dissolved in a 1:1 solution of dimethyl sulfoxide and isopropanol. The absorbance of the solution was measured at 570 nm (SpectraMax spectrophotometer; Molecular Devices, Sunnyvale, CA, USA).

#### 2.3.4. Fluorescent Viability Staining

To further assess their viability, the cells were incubated with 50 mM calcein acetoxymethyl ester (Calcein-AM, which appears green under fluorescence microscopy; Molecular Probes, Eugene, OR, USA) in a culture medium for 20 min at 37 °C; and, the stained cells were visualized under a fluorescence microscope (OLYMPUS IX71, Tokyo, Japan) at 20× magnification. Digital images were acquired with a charge-coupled device camera (Axiocam b/w; Zeiss).

#### 2.3.5. SEM

3% of glutaraldehyde was used to fix the cells for 24 h at 20 °C, dehydrated using a graded series of ethanol (25%, 50%, 70%, 80%, 90%, 95%, and 100%), dried using CO_2_ critical point drying (HCP-2; Hitachi, Tokyo, Japan), and sputter-coated with gold before SEM viewing. The butterfly wings were also characterized using a JEOL JSM-6360LV field-emission scanning electron microscope (FE-SEM), equipped with a JED2300 EDS system to confirm the composition of the butterfly wings before and after the Acidic/base treatment.

#### 2.3.6. Statistical Analysis

Experiments were repeated in duplicate or triplicate for at least 2–3 times for each condition. The data are expressed as the mean ± standard deviation. The t-test was used for the statistical analyses. A value of *p* < 0.05 was considered statistically significant. All data were analyzed with SPSS version 11.0 (SPSS, Inc., Chicago, IL, USA).

## 3. Results and Discussion 

**Figure 1:**
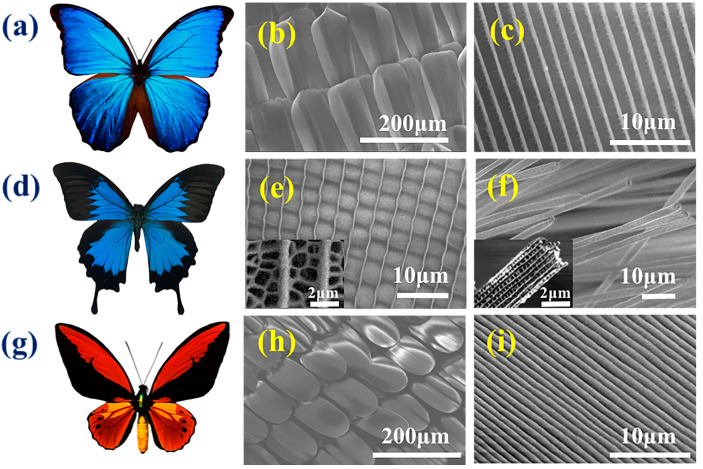
(**a**) Optical image of *M. menelaus*; (**b**,**c**) Scanning Electron Microscope (SEM) image of the nanostructure of the wing under different magnification. (**d**) Optical image of *P. u. telegonus*; (**e**) SEM image of the nanostructure of the blue region; the insert in (**e**) is the high magnification of SEM image; (**f**) SEM image of the nanostructure of the fiber region; and, the insert in (**f**) is the high magnification of SEM image. (**g**) Optical image of *O. c. lydius*; (**h**,**i**) the SEM image of the nanostructure of the wing according to different magnification.

Among the three designated species of butterflies, *M. menelaus*, which belongs to the *Morpho* genus, has been extensively studied because of its shiny blue color (Figure 1a). Generally, the bright colors are from the pigment and structural colors. The pigment color relies primarily on the absorption of light through chemical chromophores, while the structural color relies on the transportation of light concerning the photonic nanostructure units, located on the skin or surface. Figure 1b,c illustrates that the microstructure of the surface of the *M. menelaus* was periodically ordered in grooves and ridges, making it an anisotropic structure. The *P. u. telegonus*, which belongs to the *Papilio* genus, has two significantly diverse regions, the blue region and the fiber region (Figure 1d). The blue region is represented by the blue area of the wing, while the fiber region consists of the black area of the wing. It was discovered that the blue region exhibits grooves/ridge structures similar to that of anisotropic structures of *M. menelaus*. High magnification of the Scanning Electron Microscope (SEM) images clearly displayed the details of the struts in between the ridges (Figure 1e). In contrast, the fiber region appears to be constructed of another photonic microstructure, but in the form of nanofiber structure (Figure 1f). The *O. c. lydius*, which belongs to the *Ornithoptera* genus, typically has yellow and black colors (Figure 1g), and its nanostructure also consists of paralleled grooves/ridges (Figure 1h,i). Overall, these three species of butterflies are typical of their genera, and their anisotropic nanostructures, including grooves/ridges and fiber structure, are a common for in vivo cell culture substrates.

**Figure 2:**
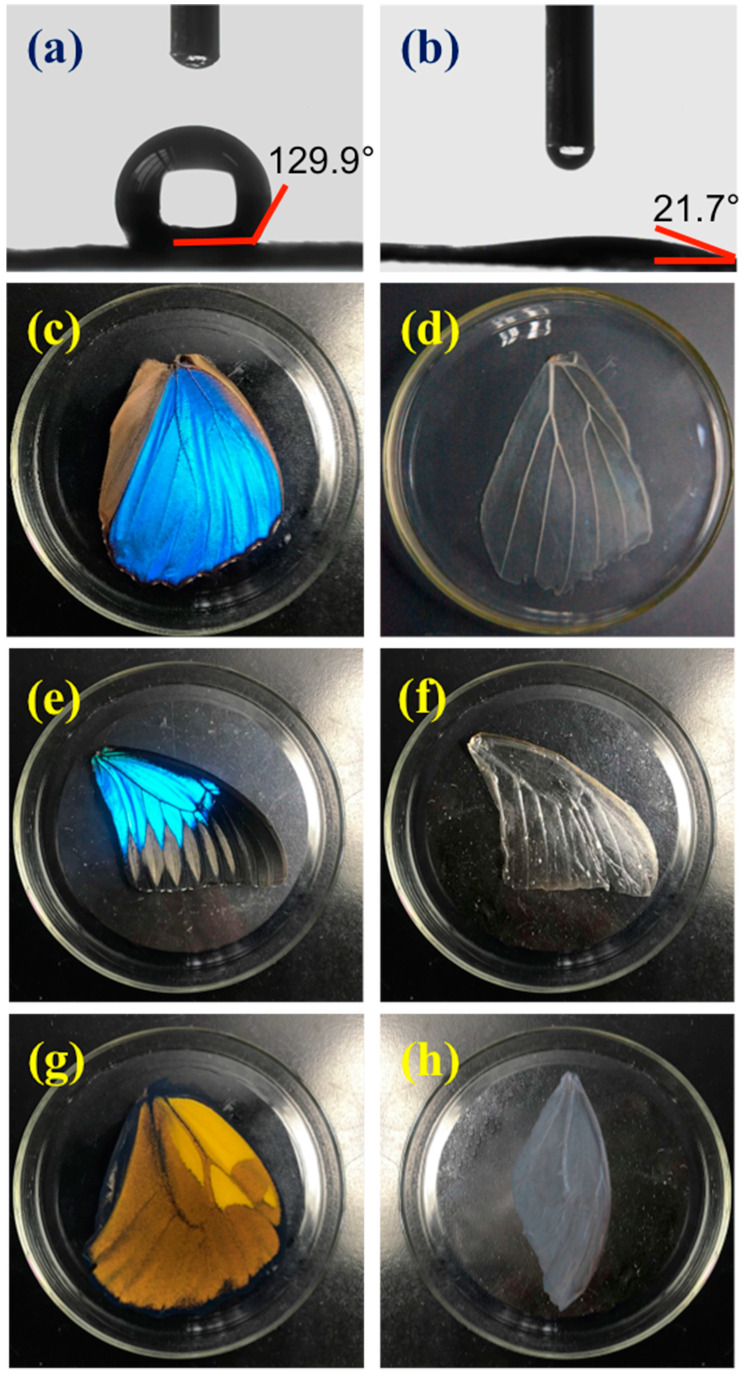
(**a**,**b**) are the water contact angle of *M. menelaus* pre and post plasma treatment. (**c**,**d**) are the optical image of *M. menelaus* wing pre and post chemical treatment. (**e**,**f**) are the optical image of *P. u. telegonus* wing pre and post chemical treatment. (**g**,**h**) are the optical image of *O. c. lydius* wing pre and post chemical treatment.

Naturally, to avoid influences from rain or other natural disasters, these butterfly wings are strongly hydrophobic on both the dorsal and ventral sides. Thus, it is challenging to conduct any experiments on those wings, since the strong hydrophobic features could hinder the contact with aqueous substrates. Moreover, these particular wings also contain chitin, proteins, and some pigment, which should be removed to obtain chitosan, a more biocompatible environment for cell culturing [51,52,53]. In a typical experiment, two steps were conducted prior to cell culture. First, a plasma treatment was conducted to generate hydroxyl (–OH), thereby rendering the wings hydrophilic. Compared to the untreated wings (Figure 2a), the plasma treated wing revealed high hydrophilicity (Figure 2b). To alter the main wing compound of chitin to chitosan, as well as to remove its pigment and other proteins that may affect cells, HCl/NaOH was subsequently applied on the wings (Figure S1, Supplementary Material). The *M. menelaus* wings turned transparent after HCl/NaOH treatment, indicating that the pigments were removed (Figure 2c,d). It was worth noting that the *M. menelaus* wings retained a slightly blue structural color, which revealed that the main structures were not destroyed during treatment. However, there was no structural color was observed in *P. u. telegonus* and *O. c. lydius* post-treatment (Figure 2e–h). This may be because the pigments contribute to the primary colour of these two types of butterfly wings when compared to *M. menelaus*.

**Figure 3:**
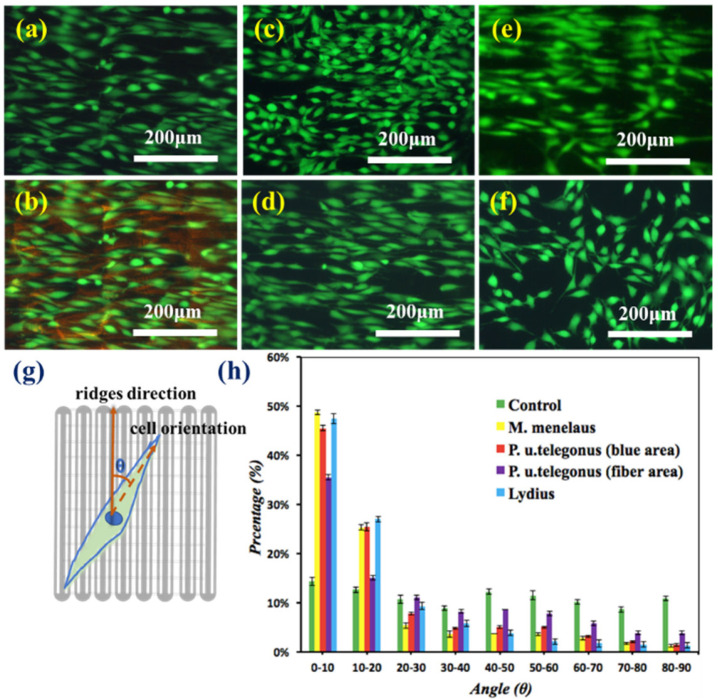
Fluorescence microscopy images of NIH–3T3 fibroblast cells cultured on different substrates after 48 h: (**a**,**b**) *M. menelaus*, (**c**) *P. u. telegonus* (blue region), (**d**) *P. u. telegonus* (fiber region), (**e**) *O. c. lydius*, and culture dish (**f**) as a control. 500 cells were measured on each substrate; (**g**) Schematic diagram of the orientation angle of the cells on the substrates, the dash line stands for the direction of cells orientation, the red arrows stand for the direction of grooves/ridges; and, (**h**) represents the frequency distribution of orientation angle of cells cultured on different substrates after 48 h. The area of 500 cells was measured on each substrate.

After sterilization, the wings were ready for cell culture. The NIH-3T3 fibroblast cells were then seeded on the surface of *M. menelaus*, *P. u. telegonus* (blue area), *P. u. telegonus* (fiber area), *O. c. lydius*, and cell culture dish at a density of 2 × 10^3^ cells/mL, respectively. Cells were then stained with a live cell viability assay, Calcein-AM for fluorescence images after 48 h of culturing. Compared with the cells on the culture dish, the cells growing on those butterfly wings showed a similar amount. It revealed that those chitinous architectures originating from butterfly wings exhibit a good biocompatibility in cell culturing [54,55,56,57,58,59]. Meanwhile, it was determined that the majority of cells revealed a certain oriented growth along the surface of *M. menelaus* (Figure 3a,b), as well as *P. u. telegonus* (either on the blue region or fiber region) (Figure 3c,d). Similar results were also found on the surface of *O. c. lydius* (Figure 3e). In comparison, cells cultured on the ordinary culture dish exhibited random orientations (Figure 3f). These results demonstrate that there is a significant impact on the alignment of cells from the nanostructure of these selected butterfly wings.

To attain a quantitative analysis of the cell orientation in response to these wings, the angles between the direction of cell orientation, and the direction of the anisotropic nanostructures were measured by ImageJ (available at http://rsbweb.nih.gov/ij/). Based on the fluorescent images, the long axis of each cell that was parallel to the direction of the grooves/ridges denoted to be 0°, while 90° represents a perpendicular direction to the grooves/didges. Figure 3g illustrates the schematic diagram of angles between the directions of cell orientation and grooves/ridges. The percentages of striated cells aligned within 0° to 90° (with incremental interval of 10°) were counted, respectively. Figure 3h illustrates the cells that showed within a 10° angle with grooves/ridges direction up to 50% of the total number on *M. menelaus*, *P. u. telegonus* (blue area) and *O. c. lydius*. Moreover, this value climbed to 90% when within a 30°angle with the grooves/ridges direction. Even on the fiber area of *P. u. telegonus*, the amount of cells that showed within a 30° angle with grooves/ridges direction up to 60% of total cells. In contrast, there was no significant trend of cell orientation distribution observed on the surface of culture dish. All of these results combined indicate that the microstructure of these selected butterfly wings could induce cell alignment effectively.

**Figure 4:**
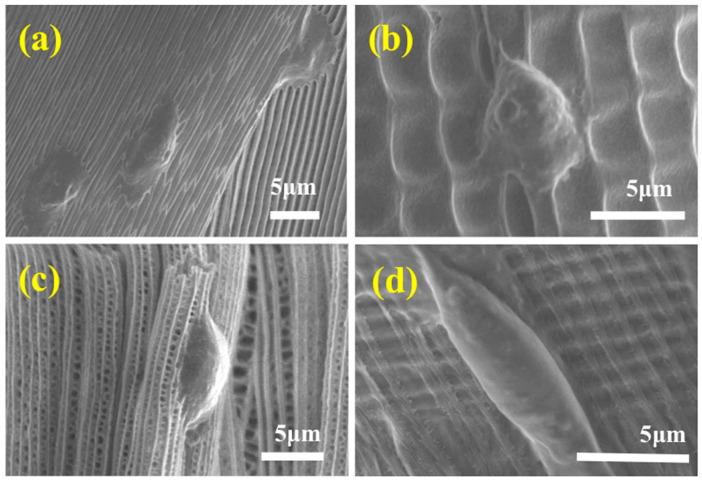
SEM images of NIH-3T3 fibroblast cells after 48 h cultured on the wing of (**a**) *M. menelaus*; (**b**) *P. u. telegonus* (blue region); (**c**) *P. u. telegonus* (fiber region), and (**d**) *O. c. lydius* *wings*.

The details of the fibroblasts elongation in each wing were also investigated by utilizing SEM (Figure 4). It was ascertained that the microstructure of each wing retained the same formation, indicating little damage to the wings during chemical treatment. It can also be observed that the fibroblast cells cultured on the surface of the *M. menelaus* displayed a distinct alignment along the direction of the grooves/ridges (Figure 4a). The same results can also be viewed on the surface of other wings (Figure 4b–d). These observations were consistent with the fluorescent results.

**Figure 5:**
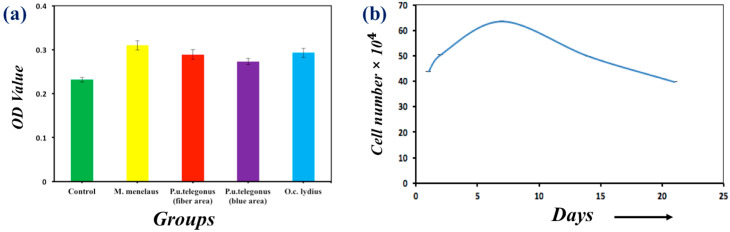
(**a**) MTT activity assay of NIH-3T3 fibroblast cells cultured on different substrates: *M. menelaus*, *P. u. telegonus* (blue area and fiber area), *O. c. lydius* and cell culture dish after 48 h; (**b**) A long period culture of NIH-3T3 fibroblast cells on an *M. menelaus* wing.

Cell viability on the wings and culture dish was also investigated quantitatively by MTT assays, as presented in Figure 5a. It was determined that the cells on each wing demonstrated a similar viability when compared to the cells cultured on culture dishes. Meanwhile, a longer culturing period of cells was also conducted on each wing to identify cell proliferation. Figure 5a shows that the population of cells cultured on the *M. menelaus* wings reached its peak amount at one week intervals and gradually decreased during the following weeks, which satisfies the stand cell proliferation curve. Similar results can also be found on other butterfly wings (Figure S2, Supplementary Material). Taken together, these results indicate that the anisotropic nanostructure of these butterfly wings could influence the alignment of fibroblast cells.

## 4. Conclusions

In conclusion, a simple, inexpensive, and green method for cell culture was presented. The cell culture substrate originated from butterfly wings with natural anisotropic nanostructures. A two-step chemical treatment was proposed to get more hydrophilic butterfly wings preceding cell culturing. Cells culturing on those substrates demonstrated a high degree of alignment along the direction of the grooves/ridges. To our knowledge, this is the first report to use naturally pre-structured chitin/chitosan scaffolds, isolated from butterfly wings as fibroblast cells culture substrates. We anticipate that those natural substrates originating from natural butterfly wings will have important applications in tissue engineering.

## Supplementary Materials

The following are available online at https://www.mdpi.com/2073-4360/9/9/386/s1, Figure S1: EDS Spectrum, Figure S2: MTT activity assay.
