# Correction: Elbaz et al. Chitin-Based Anisotropic Nanostructures of Butterfly Wings for Regulating Cells Orientation. *Polymers* 2017, *9*, 386

**DOI:** 10.3390/polym15234582

**Published:** 2023-11-30

**Authors:** Abdelrahman Elbaz, Jie Lu, Bingbing Gao, Fuyin Zheng, Zhongde Mu, Yuanjin Zhao, Zhongze Gu

**Affiliations:** 1 State Key Laboratory of Bioelectronics, School of Biological Science and Medical Engineering, Southeast University, Nanjing 210096, China; chem.egy@gmail.com (A.E.); 101101546@seu.edu.cn (J.L.); 230139435@seu.edu.cn (B.G.); 230139156@seu.edu.cn (F.Z.); 230129294@seu.edu.cn (Z.M.); yjzhao@seu.edu.cn (Y.Z.); 2 National Demonstration Center for Experimental Biomedical Engineering Education, Southeast University, Nanjing 210096, China; 3 Laboratory of Environment and Biosafety, Research Institute of Southeast University in Suzhou, Suzhou 215123, China

In the original publication [1], there was a mistake in Figure 3c,e as published. The corrected Figure 3 appears below. 

The authors state that the scientific conclusions are unaffected. This correction was approved by the Academic Editor. The original publication has also been updated.

## Figures and Tables

**Figure 3:**
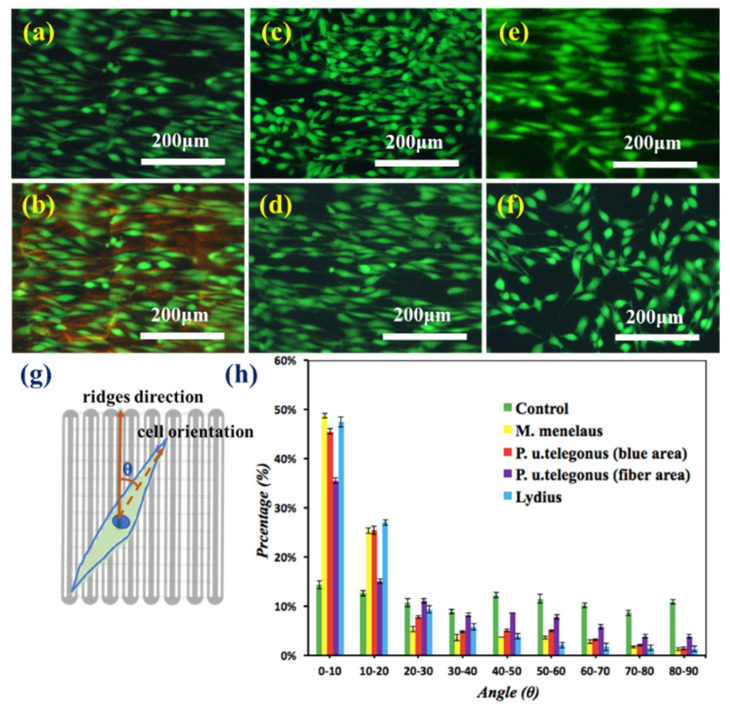
Fluorescence microscopy images of NIH–3T3 fibroblast cells cultured on different substrates after 48 h: (**a**,**b**) *M. menelaus*, (**c**) *P. u. telegonus* (blue region), (**d**) *P. u. telegonus* (fiber region), (**e**) *O. c. lydius*, and culture dish (**f**) as a control. 500 cells were measured on each substrate; (**g**) Schematic diagram of the orientation angle of the cells on the substrates, the dash line stands for the direction of cells orientation, the red arrows stand for the direction of grooves/ridges; and, (**h**) represents the frequency distribution of orientation angle of cells cultured on different substrates after 48 h. The area of 500 cells was measured on each substrate.
